# Atypical Anti-glomerular Basement Membrane Disease in a 16-Year-Old Male Child: A Case Report

**DOI:** 10.7759/cureus.78644

**Published:** 2025-02-06

**Authors:** Deepika Singh, Brian Pugmire, Sukesh Sukumaran

**Affiliations:** 1 Pediatric Rheumatology, Valley Children’s Healthcare, Madera, USA; 2 Radiology, Valley Children’s Healthcare, Madera, USA; 3 Pediatrics, Valley Children’s Healthcare, Madera, USA

**Keywords:** anti-gbm disease, anti-glomerular basement membrane antibody disease, glomerulonephritis, goodpasture disease, small vessel vasculitis

## Abstract

Anti-glomerular basement membrane (anti-GBM) disease is an extremely rare small vessel vasculitis, which typically presents as rapidly progressive glomerulonephritis with or without pulmonary hemorrhage. Atypical anti-GBM disease varies in its clinical and laboratory presentation with insidious onset of symptoms. We present the case of a 16-year-old male child who presented with a two-week history of fever, weight loss, cough, hemoptysis, shortness of breath, and a five-year history of intermittent emesis. A computerized tomography of the chest demonstrated diffuse miliary pulmonary nodules with a "tree-in-bud" pattern. Serologic evaluation was negative for anti-nuclear, double-stranded, and anti-neutrophilic cytoplasmic antibodies. Urinalysis was negative for hematuria and proteinuria, but anti-GBM antibodies were elevated. Kidney biopsy demonstrated linear immunofluorescence staining of glomerular basement membrane with immunoglobulin G (IgG) without active crescent formation or necrosis. Lung biopsy demonstrated occasional hemosiderin-laden macrophages, patchy peribronchial and interstitial lymphocytic inflammation, interstitial and alveolar septal fibrosis, and emphysema. The patient was diagnosed with atypical anti-GBM disease based on the circulating and tissue-bound antibodies on kidney and lung biopsy and chronic alveolar bleeding, and improved with treatment with intravenous steroids, cyclophosphamide, and rituximab. This case report highlights the importance of a high index of suspicion for this disease and the need to perform a renal biopsy even in the absence of hematuria or proteinuria. Additionally, this case was unusual as the patient presented primarily with pulmonary and gastrointestinal symptoms and normal renal functions. His pathology was limited to linear immunofluorescence without active crescent formation, and this has not been previously reported, to our knowledge.

## Introduction

Anti-glomerular basement membrane (anti-GBM) disease, previously known as Goodpasture disease, is a rare small vessel vasculitis characterized by the deposition of circulating anti-GBM antibodies in the kidney and lung tissue. Although estimating precise incidence is challenging due to the rare nature of the condition, anti-GBM disease is estimated to account for 0.4-3% of pediatric crescentic glomerulonephritis [1,2]. Children with classic (anti-GBM) disease present with rapidly progressive glomerulonephritis, life-threatening hemoptysis, or a combination of both. Anti-GBM disease is caused by immunoglobulin G (IgG) autoantibodies that deposit and attack the non-collagenous domain of the alpha 3 chains of type IV collagen found in the kidney basement membrane, lungs, cochlea, and retina [3]. Renal histopathology demonstrates linear immunofluorescent staining for IgG on the glomerular basement membrane, causing crescentic glomerulonephritis, and enzyme-linked immunosorbent assay (ELISA) detects circulating anti-GBM antibodies [2].

Atypical anti-GBM disease is a variant of anti-GBM disease and patients present with unique clinical and pathological findings. It comprises only 8-12% of anti-GBM cases in the reported literature [4-8]. In contrast to the classic anti-GBM disease, the presentation of atypical anti-GBM is more indolent, and patients exhibit milder symptoms. Additionally, pulmonary hemorrhage is less common than in clinical anti-GBM disease, and renal outcomes tend to be more favorable. Patients have nephrotic range proteinuria, with a range of mild to typical severe outcomes [9,10]. Patients with atypical anti-GBM disease also lack circulating serum anti-GBM IgG antibodies [5-7,11], and this is thought to be related to the presence of pathogenic immunoglobulins that recognize different anti-GBM epitopes than classic anti-GBM disease [5,6]. Most of these cases are described in adults, with only a few cases reported in children [10,12]. Patients with anti-GBM disease require aggressive therapy with corticosteroids, cyclophosphamide, and plasma exchange [13]. Still, in contrast, limited data exists on how to treat atypical anti-GBM disease.

Here, we present the case of an adolescent male with pulmonary and gastrointestinal symptoms but normal renal function. However, serum anti-GBM antibodies were present, and kidney biopsy demonstrated linear immunofluorescence staining of glomerular basement membrane with IgG without active crescent formation or necrosis. He achieved remission with treatment with high-dose corticosteroids, cyclophosphamide, and rituximab.

## Case presentation

A 16-year-old, previously healthy, male child presented to the emergency department with a two-week history of fever, weight loss, cough, hemoptysis, shortness of breath, and intermittent non-bilious, non-bloody emesis. His past medical history, surgical history, and family history were non-contributory. The family denied any history of travel or animal exposure. He denied any sick contacts, ingestion of unpasteurized dairy products, or tuberculosis exposures. He had no history of recurrent infections. The patient denied smoking or vaping but reported dust exposure and resided in the Central Valley of California.

Physical examination revealed an ill-appearing child. His temperature was 38°C, pulse 101 bpm, blood pressure 116/77 mm mercury, respiratory rate 44 breaths per minute, and pulse oximetry 90% on room air. The patient was in moderate respiratory distress, and auscultation of his lungs revealed decreased breath sounds with diffuse crackles bilaterally. The remainder of his examination was within normal limits. Initial laboratory evaluation showed a normal complete blood count with differential and a complete metabolic panel notable for normal blood urea nitrogen (12 mg/dL) and creatinine (0.88 mg/dL) (Table 1).

**Table 1:** Laboratory values at initial presentation BUN: blood urea nitrogen; AST: aspartate aminotransferase; ALT: alanine transaminase; CRP: C-reactive protein; LDH: lactate dehydrogenase; MCV: mean corpuscular volume; MCH: mean corpuscular volume; MCHC: mean corpuscular hemoglobin concentration; RDW: red cell distribution width; MPV: mean platelet volume

Laboratory parameter	Patient value	Reference range and units
Sodium	139 mEq/L	136-145 mEq/L
Potassium	4.4 mEq/L	3.5-5.0 mEq/L
Chloride	104 mEq/L	98-106 mEq/L
Total CO2	23 mEq/L	23-28 mEq/L
BUN	12 mg/dL	8-20 mg/dL
Creatinine	0.88 mg/dL	Male: 0.7-1.2mg/dL; Female: 0.5-1.0 mg/dL
Glucose	90 mg/dL	70-105 mg/dL
Calcium	9.4 mg/dL	9-10.5 mg/dL
Alkaline Phosphatase	100 U/L	36-150 U/L
Albumin	4.0 g/dL	3.5-5.4 g/dL
Total Protein	7.1 g/dL	6-7.8 g/dL
AST	20 U/L	<35 U/L
ALT	36 U/L	<35 U/L
Bilirubin, Conjugated	0.30 mg/dL	0-0.3 mg/dL
Bilirubin, Unconjugated	0.2 mg/dL	0.2-0.8 mg/dL
Bilirubin, Total	0.50 mg/dL	0.3-1.2 mg/dL
Lipase	19 U/L	<95 U/L
CRP	8.6 mg/dL	<0.8 mg/dL
Ferritin	228.3 mg/mL	Male: 30-300 ng/mL; Female: 30-200 ng/mL
LDH	139 U/L	60-160 U/L
Triglyceride	88 mg/dL	<150 mg/dL
Procalcitonin	0.05 μg/L	<0.1 μg/L
White Blood Cells	12.9 x 10^3 cells/ mcL	4.5-11 x 10^3^ cells/ mcL
Red Blood Cells	4.69 x 10^6 cells/ mcL	4.2-5.9 x 10^6^ cells/ mcL
Hemoglobin	14.4 g/dL	Male: 14-17 g/dL Female: 12-16 g/dL
Hematocrit	42.0%	Male: 40-54%; Female: 36-48%
MCV	89.7 fL	80-100 fL
MCH	30.8 pg	28-32 pg
MCHC	34.4 g/dL	32-36 g/dL
RDW	12.6%	Male: 11.8-14.5%; Female: 12.2-16.1%
Platelet	373 x10^3/mcL	150-350 x 10^3^/mcL
Neutrophils, % Auto	80.2 %	43-76%
Lymphocyte, % Auto	7.8 %	8-41%
Monocyte, % Auto	8.8 %	4-8%
Eosinophil, % Auto	2.4 %	2-4%
Basophil, % Auto	0.7 %	0-1%

**Figure 1:**
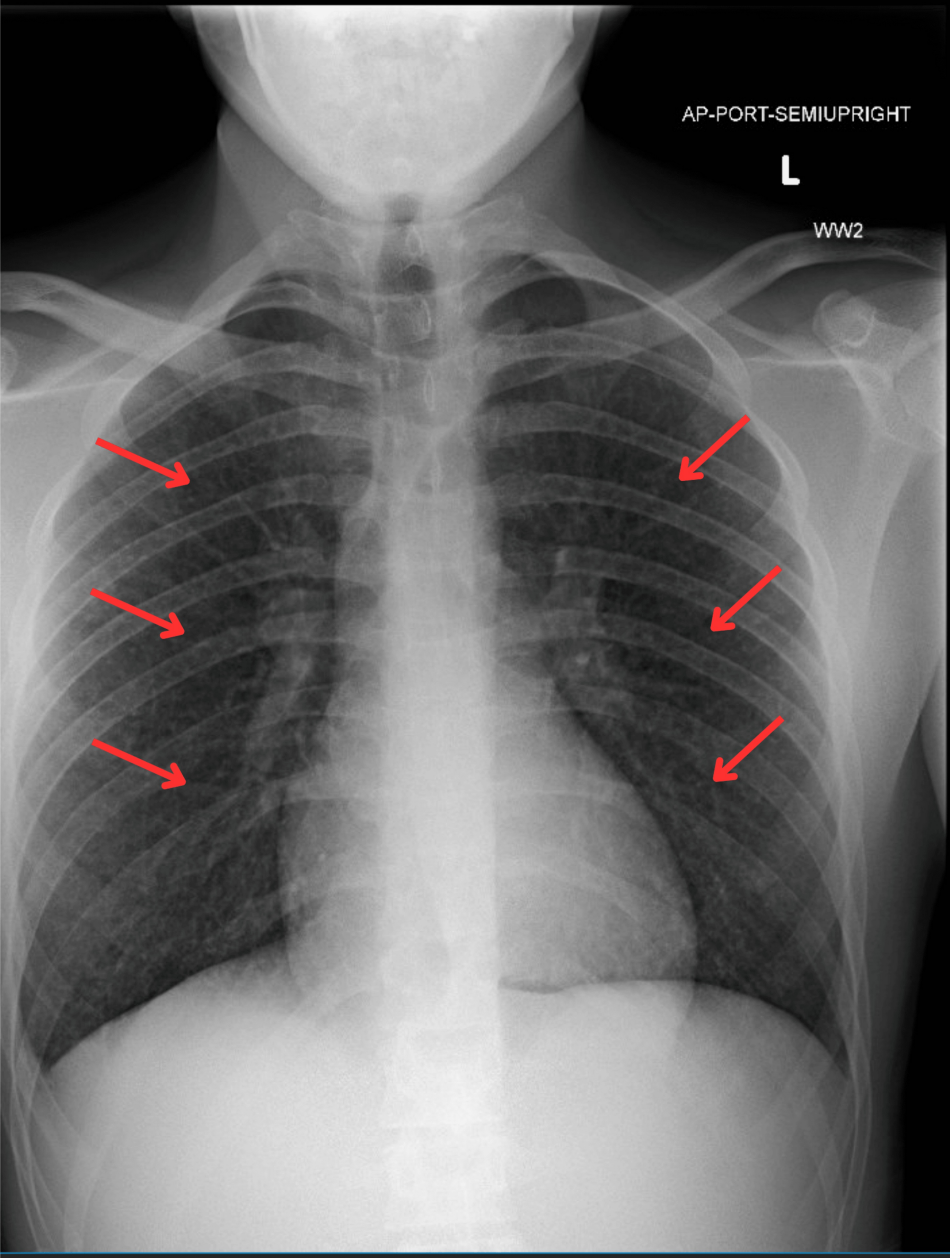
Chest X-ray demonstrating reticular opacities (red arrows) in bilateral lung fields

**Figure 2:**
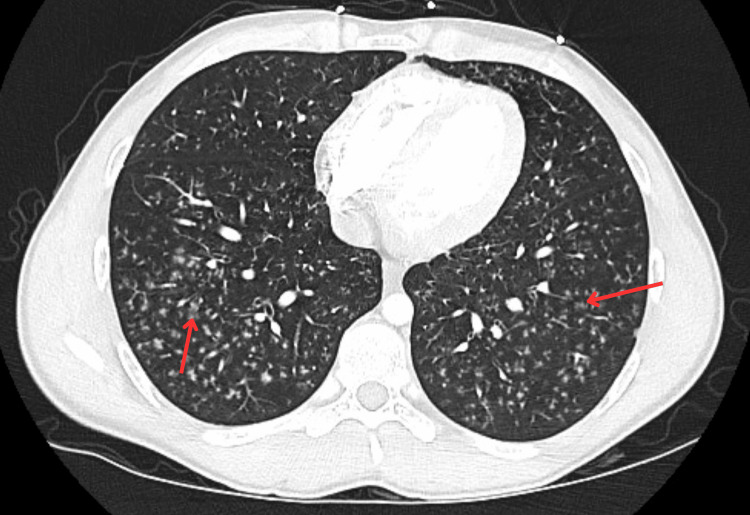
CT chest demonstrating diffuse miliary pulmonary nodules with a "tree-in-bud" pattern (red arrows)

Erythrocyte sedimentation rate (23 mm/hour), and C-reactive protein (8.6mg/dL) were elevated. His C3 and C4 also elevated at 175.4 mg/dL and 37.8 mg/dL, respectively (normal range C3 83-152 mg/dL; C4 13-37 mg/dL). Chest X-ray demonstrated reticular opacities in bilateral lung fields (Figure 1). Computed tomography (CT) of the chest showed diffuse miliary pulmonary nodules with a "tree-in-bud" pattern (Figure 2).

Autoimmune serology included negative antinuclear antibody (ANA), anti-double-stranded DNA, and antineutrophil cytoplasmic antibody titers (ANCA). Anti-GBM antibodies were positive (62, normal range 0-19 AU/ml). Urinalysis demonstrated specific gravity of 1.013, WBC 2 (normal range 0-28), RBC 3 (normal range 0-22), negative for leukocyte esterase, nitrites, protein, glucose, and blood casts. The urine toxicology screen was positive for marijuana.

He was started on ceftriaxone, vancomycin, and azithromycin and admitted to the pediatric intensive care unit due to persistent hemoptysis and deteriorating respiratory status.

An infectious workup was negative for coccidioidomycosis, *Cryptococcus*, *Blastomyces*, *Histoplasma*, and human immunodeficiency virus. His QuantiFERON-TB Gold test (QIAGEN N.V., Venlo, Netherlands) was negative. Bronchoscopy revealed normal-appearing mucosa and no active pulmonary bleeding. Bronchioalveolar lavage studies were negative for an infectious process. Esophagogastroduodenoscopy (EGD) demonstrated no evidence of mucosal disease with normal histology.

**Figure 3:**
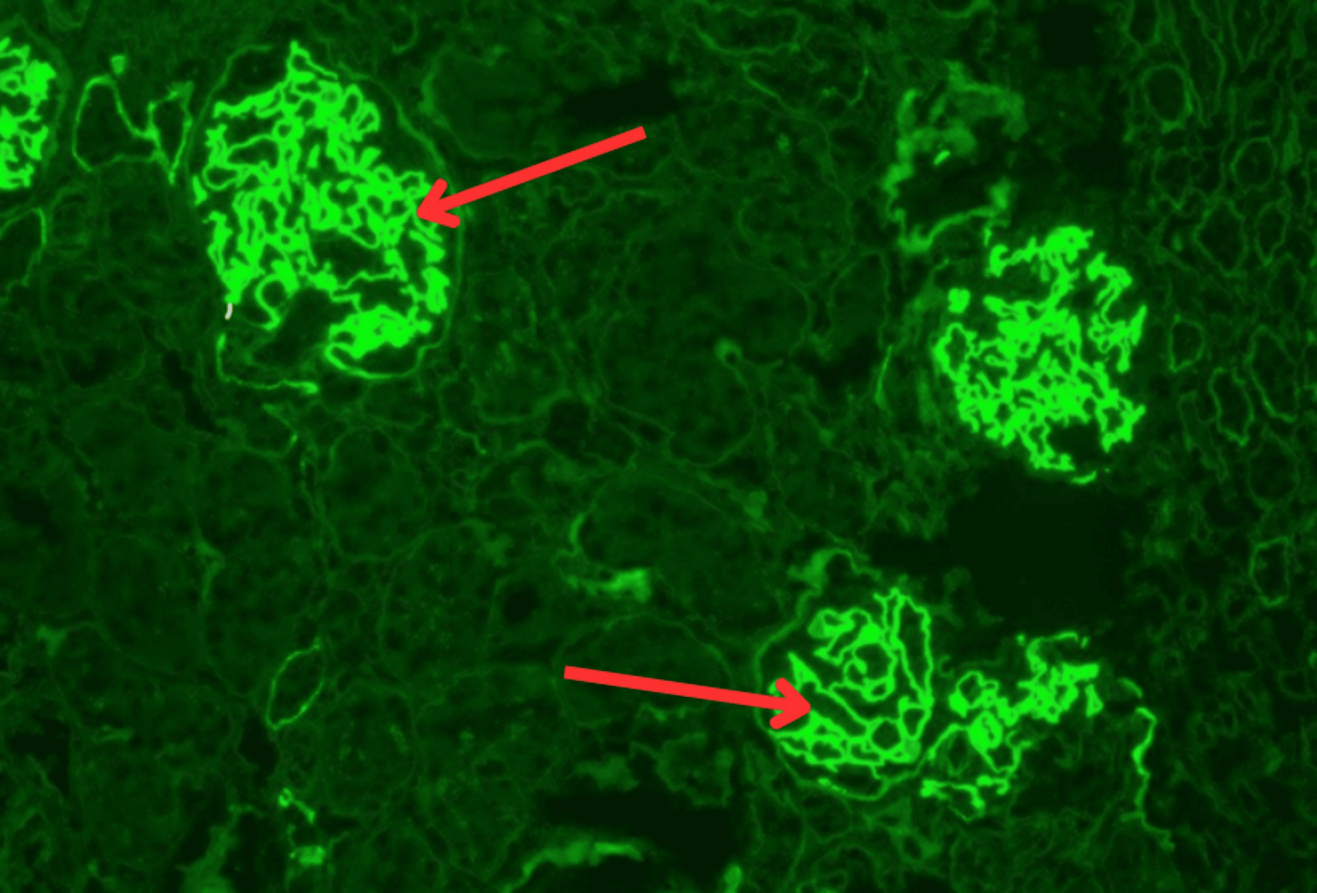
Examination under immunofluorescence microscopy demonstrating linear immunoglobulin G (IgG) staining of the glomerular basement membrane (red arrows)

Due to concern for anti-GBM disease, tissue biopsies were obtained. Kidney biopsy demonstrated strong and diffuse linear staining for IgG (4+), kappa (2+), and lambda (3-4+) light chains (Figure 3).

**Figure 4:**
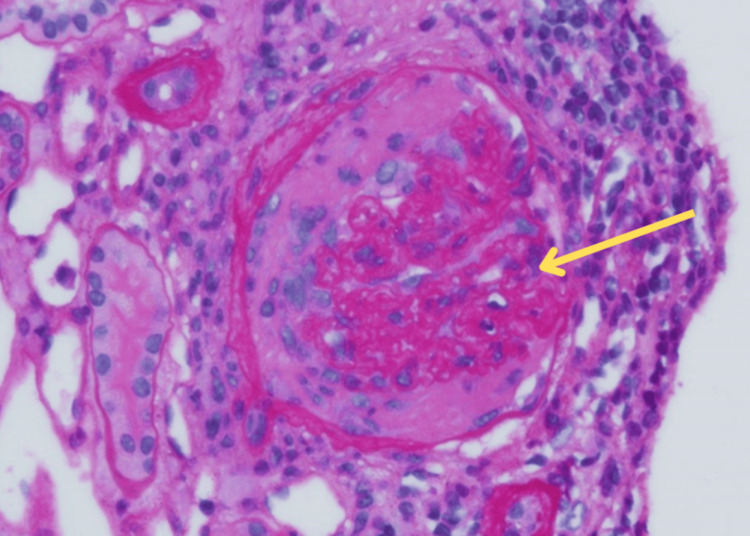
Light microscopic examination of renal biopsy with hematoxylin-eosin stain demonstrating rare remote fibrous crescent (yellow arrow)

Mesangial areas stained for IgM trace in a finely granular pattern and arteriole walls stained for C3 (3+). There was no active crescent formation or necrosis. Two remote/fibrous crescents were seen, possibly related to anti-GBM antibodies (Figure 4).

There was no significant parenchymal scarring. Lung biopsy demonstrated occasional intra-alveolar and interstitial hemosiderin-laden macrophages. Patchy peribronchial and interstitial lymphocytic inflammation was seen along with interstitial and alveolar septal fibrosis and emphysema. Capillaritis was not seen on lung biopsy.

**Figure 5:**
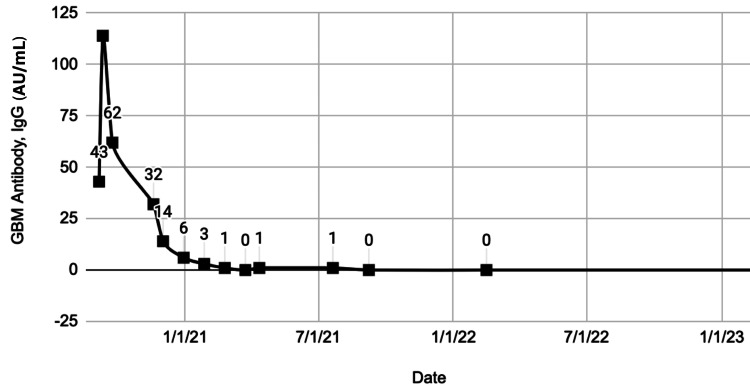
GBM antibody, IgG (AU/mL) by multiplex bead assay at diagnosis and over the course of treatment.

Given his overall clinical presentation with circulating and tissue-bound antibodies seen on kidney biopsy along with histopathological changes seen on pulmonary tissue with chronic alveolar bleeding, the patient was diagnosed with atypical anti-GBM disease. Treatment was initiated with intravenous pulse methylprednisolone and intravenous cyclophosphamide 1000 mg every four weeks for six doses and rituximab 750 mg/m^2^ for two doses. Subsequently, he was started on maintenance therapy with rituximab infusions every six months. Anti-GBM antibodies resolved with treatment and have remained in remission as of his four-year follow-up (Figure 5).

## Discussion

Our patient’s presentation of pulmonary and gastrointestinal symptoms in the absence of acute kidney injury is unique in anti-GBM disease. Secondly, kidney biopsy was crucial in establishing our patient’s diagnosis as he had normal renal function but with findings of linear immunofluorescence staining of GBM by IgG on biopsy. Finally, our patient had an excellent response to treatment with cyclophosphamide and rituximab, without any significant complications.

Anti-GBM disease is an uncommon entity with an estimated incidence of one per million per year in adults and even more rare in children [4]. With approximately 90% of patients developing rapidly progressive glomerulonephritis, early diagnosis and prompt treatment are important to prevent long-term morbidity and mortality. Strong linear immunofluorescent staining for IgG on the glomerular basement membrane is characteristic of anti-GBM disease and typically manifests with necrotizing and crescentic glomerulonephritis [2,3].

Atypical anti-GBM disease has been variably defined in the literature, with some authors using this term to define patients with necrotizing glomerulonephritis with classic histopathologic findings but with negative circulating antibodies. In other cases, patients displaying less severe symptoms and indolent course, but still with detectable antibodies have been diagnosed with atypical anti-GBM disease [8]. There have been several case studies that report unaffected renal function in patients diagnosed with atypical anti-GBM disease [14,15]. Patients with predominantly pulmonary involvement have also been described with normal renal function [16].

It is unclear if this was due to pulmonary symptoms such as hemoptysis preceding more extensive renal involvement and thus acting as a hint towards earlier diagnosis or presenting a unique subset of patients. Pediatric cases, both of typical and atypical anti-GBM, are extremely rare.

Young male patients and those who smoke are more likely to present with both pulmonary and renal symptoms in anti-GBM disease whereas older patients more frequently present with renal disease [17]. Our patient presented with predominantly respiratory symptoms without overt renal manifestations. Interestingly, military pulmonary nodules have not been previously described in the context of anti-GBM disease. Studies have indicated that exposure to smoking, and organic solvents may be a risk factor for pulmonary anti-GBM disease [2,16]. This is likely due to the increased lung capillary permeability that occurs during smoking, which may allow circulating anti-GBM antibodies to attach to the alveolar basement membrane more easily. Additionally, smoking and inhaling hydrocarbon and organic solvents may directly injure the alveolar basement membrane, further inducing antibody formation [14]. While our patient tested positive for marijuana, he denied any smoking or known secondhand smoking exposure or nicotine use. He resides in California’s Central Valley, an area with exceptionally poor air quality due to geography and use of pesticides in this agricultural community [18], and it is unclear if this was a contributory factor. 

Along with multiorgan involvement, pediatric patients are more likely to have more detrimental kidney involvement with crescent formation and scarring.

Treatment

The vast majority of patients with initial renal impairment in anti-GBM disease will progress to lifelong renal difficulties, often resulting in renal failure [19]. Thus, it is critical that patients are properly diagnosed quickly and treated aggressively to avoid recurrence of antibody formation and to preserve remaining kidney function.

Kidney Disease Improving Global Outcomes (KDIGO) guidelines recommend rapid, aggressive treatment with immunosuppressive agents such as cyclophosphamide and glucocorticoids in conjunction with plasmapheresis in all patients except those treated initially with dialysis, have extensive glomerulosclerosis or 100% crescent formation on biopsy, and are without pulmonary hemorrhage [13]. However, there are no current established national guidelines for pediatric-specific treatment due to its rarity in this population. 

Our patient tolerated both cyclophosphamide and rituximab therapy well and continues to have normal renal functions, four years after initial diagnosis. Although many patients described in the literature [20,21] have progressed to renal failure even with aggressive immunosuppressive therapy, the patient in the current report appears to be in a unique category of those who perform well. Few cases with similar positive outcomes have been described in the literature. Another case of positive renal prognosis was described in a case study by Dixit et al., in which they detail the nearly complete return of normal renal function in a three-year-old child with anti-GBM disease three years after diagnosis and aggressive treatment [19]. Unfortunately, early and aggressive treatment may not be sufficient in all patients and there is still a need for research to determine factors responsible for poor prognosis.

We hypothesize that the reason our patient has done so well is due to a possibly a combination of early diagnosis prior to the development of renal manifestations and a milder form of the disease, supported by his lack of crescentic glomerular formation and lack of other significant renal or autoimmune comorbidities.

Currently, treatment approaches for pediatric anti-GBM disease are extrapolated from adult patients. Several case studies have demonstrated that aggressive treatment has improved long-term renal function in many patients and helped both quickly remove anti-GBM antibodies and avoid further development [13,14,19]. Thus far, plasmapheresis has been the mainstay of treatment for classic anti-GBM disease to quickly halt the onslaught of damage by anti-GBM antibodies. However, in our patient’s case, we decided against plasmapheresis, given the milder presentation and the potential risk of complications associated with plasmapheresis in the context of his overall healthy appearance. While untreated anti-GBM disease will progress to kidney failure with high levels of mortality, the possible side effects of aggressive immunosuppressive treatment including high doses and prolonged courses of steroids and chemotherapeutic agents and plasmapheresis need to be closely monitored. Further research is needed to establish and optimize the treatment of anti-GBM disease in the pediatric population.

## Conclusions

Anti-GBM disease is a rare condition with relatively few cases of atypical anti-GBM represented in pediatric literature. Our patient was unique in that he presented with normal renal functions in the setting of predominantly respiratory and gastrointestinal symptoms rather than the classic rapidly progressive glomerulonephritis. Clinicians should have a high index of suspicion for atypical anti-GBM disease when evaluating children with hemoptysis or renal disease. Renal biopsy is crucial in the diagnosis of anti-GBM disease, especially in cases with a high index of suspicion. The optimal treatment of children with atypical anti-GBM remains unclear. Further studies are needed to fully characterize its pathophysiology and associated clinical outcomes in children.

## References

[1] Dowsett T, Oni L (2022). Anti-glomerular basement membrane disease in children: a brief overview. Pediatr Nephrol.

[2] Williamson SR, Phillips CL, Andreoli SP, Nailescu C (2011). A 25-year experience with pediatric anti-glomerular basement membrane disease. Pediatr Nephrol.

[3] Kalluri R, Wilson CB, Weber M, Gunwar S, Chonko AM, Neilson EG, Hudson BG (1995). Identification of the alpha 3 chain of type IV collagen as the common autoantigen in antibasement membrane disease and Goodpasture syndrome. J Am Soc Nephrol.

[4] McAdoo SP, Pusey CD (2017). Anti-glomerular basement membrane disease. Clin J Am Soc Nephrol.

[5] Bharati J, Yang Y, Sharma P, Jhaveri KD (2023). Atypical anti-glomerular basement membrane disease. Kidney Int Rep.

[6] Ohlsson S, Herlitz H, Lundberg S, Selga D, Mölne J, Wieslander J, Segelmark M (2014). Circulating anti-glomerular basement membrane antibodies with predominance of subclass IgG4 and false-negative immunoassay test results in anti-glomerular basement membrane disease. Am J Kidney Dis.

[7] Nasr SH, Collins AB, Alexander MP (2016). The clinicopathologic characteristics and outcome of atypical anti-glomerular basement membrane nephritis. Kidney Int.

[8] Troxell ML, Houghton DC (2016). Atypical anti-glomerular basement membrane disease. Clin Kidney J.

[9] Elshirbeny M, Alkadi MM, Mujeeb I, Fituri O (2020). Atypical anti-glomerular basement membrane disease with diffuse crescentic membranoproliferative glomerulonephritis: case report and review of literature. Qatar Med J.

[10] Nagano C, Goto Y, Kasahara K, Kuroyanagi Y (2015). Case report: anti-glomerular basement membrane antibody disease with normal renal function. BMC Nephrol.

[11] Tamura R, Doi T, Hirashio S, Sasaki K, Masuda Y, Shimizu A, Masaki T (2022). A case report of atypical anti-glomerular basement membrane disease. BMC Nephrol.

[12] Jen KY, Auron A (2021). Atypical antiglomerular basement membrane disease in a pediatric patient successfully treated with rituximab. Case Rep Nephrol.

[13] (2021). KDIGO 2021 clinical practice guideline for the management of glomerular diseases. Kidney Int.

[14] Cui Z, Zhao MH, Singh AK, Wang HY (2007). Antiglomerular basement membrane disease with normal renal function. Kidney Int.

[15] Ang C, Savige J, Dawborn J, Miach P, Heale W, Clarke B, Sinclair RS (1998). Anti-glomerular basement membrane (GBM)-antibody-mediated disease with normal renal function. Nephrol Dial Transplant.

[16] Lazor R, Bigay-Gamé L, Cottin V, Cadranel J, Decaux O, Fellrath JM, Cordier JF (2007). Alveolar hemorrhage in anti-basement membrane antibody disease: a series of 28 cases. Medicine (Baltimore).

[17] Beckwith H, Lightstone L, McAdoo S (2022). Sex and gender in glomerular disease. Semin Nephrol.

[18] Cisneros R, Brown P, Cameron L (2017). Understanding public views about air quality and air pollution sources in the San Joaquin Valley, California. J Environ Public Health.

[19] Dixit MP, Kirschner R, Bulimbasic S, Dixit NM, Harris A (2010). Rescue of renal function in a 3-year-old girl with Goodpasture's syndrome with a brief review of literature. NDT Plus.

[20] Glassock RJ (2023). Estimating prognosis in anti-glomerular basement membrane disease. J Am Soc Nephrol.

[21] Bharati J, Jhaveri KD, Salama AD, Oni L (2024). Anti-glomerular basement membrane disease: recent updates. Adv Kidney Dis Health.

